# Mesenchymal Stem Cell Responses to Bone-Mimetic Electrospun Matrices Composed of Polycaprolactone, Collagen I and Nanoparticulate Hydroxyapatite

**DOI:** 10.1371/journal.pone.0016813

**Published:** 2011-02-08

**Authors:** Matthew C. Phipps, William C. Clem, Shane A. Catledge, Yuanyuan Xu, Kristin M. Hennessy, Vinoy Thomas, Michael J. Jablonsky, Shafiul Chowdhury, Andrei V. Stanishevsky, Yogesh K. Vohra, Susan L. Bellis

**Affiliations:** 1 Department of Physiology and Biophysics, University of Alabama at Birmingham, Birmingham, Alabama, United States of America; 2 Center for Nanoscale Materials and Biointegration, University of Alabama at Birmingham, Birmingham, Alabama, United States of America; 3 Department of Biomedical Engineering, University of Alabama at Birmingham, Birmingham, Alabama, United States of America; 4 Department of Physics, University of Alabama at Birmingham, Birmingham, Alabama, United States of America; 5 Department of Chemistry, University of Alabama at Birmingham, Birmingham, Alabama, United States of America; University of Minho, Portugal

## Abstract

The performance of biomaterials designed for bone repair depends, in part, on the ability of the material to support the adhesion and survival of mesenchymal stem cells (MSCs). In this study, a nanofibrous bone-mimicking scaffold was electrospun from a mixture of polycaprolactone (PCL), collagen I, and hydroxyapatite (HA) nanoparticles with a dry weight ratio of 50/30/20 respectively (PCL/col/HA). The cytocompatibility of this tri-component scaffold was compared with three other scaffold formulations: 100% PCL (PCL), 100% collagen I (col), and a bi-component scaffold containing 80% PCL/20% HA (PCL/HA). Scanning electron microscopy, fluorescent live cell imaging, and MTS assays showed that MSCs adhered to the PCL, PCL/HA and PCL/col/HA scaffolds, however more rapid cell spreading and significantly greater cell proliferation was observed for MSCs on the tri-component bone-mimetic scaffolds. In contrast, the col scaffolds did not support cell spreading or survival, possibly due to the low tensile modulus of this material. PCL/col/HA scaffolds adsorbed a substantially greater quantity of the adhesive proteins, fibronectin and vitronectin, than PCL or PCL/HA following *in vitro* exposure to serum, or placement into rat tibiae, which may have contributed to the favorable cell responses to the tri-component substrates. In addition, cells seeded onto PCL/col/HA scaffolds showed markedly increased levels of phosphorylated FAK, a marker of integrin activation and a signaling molecule known to be important for directing cell survival and osteoblastic differentiation. Collectively these results suggest that electrospun bone-mimetic matrices serve as promising degradable substrates for bone regenerative applications.

## Introduction

Bone is the second most transplanted tissue in the body (after blood transfusions). Autografting of bone is extensively employed in orthopedic and dental surgeries; however the harvesting of the patient's own bone requires a second surgery that can greatly increase the time and cost for the procedure. Additionally, nonunion at the repair site is a common problem, and iliac crest harvest can lead to complications in as many as 20% of patients [1], [2], [3]. Another limitation is that the supply of bone material from the iliac crest may be inadequate when a large amount of graft material is required [4]. For these reasons, there is an immediate need for a biomaterial that can either substitute for autografted bone or serve as a temporary matrix that induces regeneration of native bone at implant sites.

It is hypothesized that the most successful biomaterials for bone repair will be those that mimic the natural extracellular matrix, thereby minimizing foreign body or fibrotic responses. Mature bone matrix is composed of 65% mineral and 35% protein. The mineral phase is a calcium phosphate mixture that is predominantly hydroxyapatite (HA). The organic phase consists of 90% collagen I fibers, and the remaining 10% is composed of various proteoglycans and other proteins [5]. Many investigators have attempted to model the natural matrix by producing materials containing HA [6], [7], [8], [9] and/or collagen I [10], [11], [12], [13], and in vitro studies suggest that these matrices are usually highly osteoconductive [14], [15]. There are currently several commercial products that utilize collagen in combination with other molecules, such as growth factors, to stimulate or guide bone regeneration. However, in order to prevent rapid degradation, these collagen-based materials must be cross-linked, which unfortunately has some disadvantages [16]. First, the use of chemical cross-linking agents, such as glutaraldehyde, has been shown to produce prolonged toxic effects [17]. In addition, cross-linking collagen biomaterials greatly reduces the average pore size, delaying vascularization of the biomaterial and the tissue in-growth necessary for complete healing [18]. As an alternative to cross-linking, combining collagen with a synthetic polymer such as polycaprolactone (PCL) can be used to improve the mechanical properties. PCL is a semicrystalline, aliphatic polyester that has a much lower rate of degradation than collagen, and is useful in a composite scaffold for increasing mechanical strength, and fine-tuning the rate of resorbability [19], [20], [21].

Electrospinning is a particularly promising technique for synthesizing biomimetic matrices [22], [23], [24], [25], [26]. With this approach, scaffolds can be produced with nanoscale fibers that mimic the size and arrangement of native collagen fibers [27]. Additionally, electrospun scaffolds have a high surface to volume ratio, and interconnecting pores, which facilitate cell adhesion and formation of cell-cell junctions. In a prior study we described the synthesis and characterization of a tri-component electrospun scaffold composed of PCL, collagen I, and nanoparticulate HA [28]. The average fiber diameter of the scaffold was 180±50 nm, which approximates the collagen fiber bundle diameter characteristic of the native extracellular matrix of bone [29]. Moreover, a uniform dispersion of nanoscale HA particles along the fiber length was observed, with only minor agglomeration. Due to problems with agglomeration, many groups have alternately explored deposition of an HA layer onto the surface of electrospun scaffolds. One benefit of electrospinning HA along with PCL and collagen I is that the presence of HA nanoparticles throughout the scaffold provides a continuous bone-like matrix to cells as the scaffold degrades *in vivo*. In the current investigation we evaluated mesenchymal stem cell (MSC) responses to the bone-like tri-component PCL/col/HA scaffolds (50%PCL/30%col I/20% HA), in direct comparison with three other scaffold formulations; 100% PCL, 100% collagen I, and a PCL/HA composite (80%PCL/20%HA).

## Materials and Methods

### Preparation of electrospun scaffolds

Four types of scaffolds were produced by electrospinning as described previously [28]: (1) 100% PCL, (2) 80wt% PCL +20wt% HA, (3) 50wt% PCL +30wt% collagen I +20wt% HA, and (4) 100% collagen I. Solutions were made by adding hexafluoroisopropanol (HFP, Sigma-Aldrich) to each mixture such that the solid weight was 7.5% of the total solution weight. PCL (MW = 110,000 Da) was purchased from Birmingham Polymers, lyophilized calf skin collagen I was from MP Biomedicals, and HA nanoparticles (10–50 nm particle size) were synthesized as described [28]. The solutions were magnetically stirred at room temperature for 1 h before loading into disposable syringes. Voltages between 15 and 25 kV were applied using a high-voltage power supply (Gamma High Voltage Research, Ormond Beach, FL). Higher voltages were found to be necessary in order to effectively spin the collagen-based mixtures without fiber beading. The grounded aluminum collection foil was located 12 cm from the tip of the electrically charged 27-gauge needle. A syringe pump was used to feed polymer solution into the needle at a feed rate of 2 mL/h. The resulting samples were randomly arranged fibers deposited as a sheet with estimated thickness between 50 and 100 µm. No chemical or radiation-induced cross-linking of PCL or collagen fibers was performed.

Following electrospinning, samples were placed under vacuum for 48 hours to remove the residual HFP solvent. To quantify residual HFP, a 25 mg sample of each scaffold was dissolved in 1 mL of deuterated chloroform (Cambridge Isotope, Andover, MA). ^19^F NMR data were collected on a Bruker DRX400 spectrometer at ambient temperature with the following parameters: 30° pulse width, 64 scans, 100 ppm spectral width, 4.6 second recycle time. A 1.0 Hz line broadening was applied before the Fourier transform. The results were compared to a standard of 10 ppm HFP in d-chloroform, and it was found that following the 48-hour vacuuming step, the amount of HFP was below the limit of detection (<1 ppm).

### Tensile testing of scaffolds

The bulk tensile properties of each scaffold formulation were determined under wet and dry conditions. The scaffolds were cut into rectangular strips (50 mm×6 mm). The thickness of the fibrous specimen was measured at 3 different positions and the average thickness was taken to calculate the cross sectional area of the specimen. Each sample (n = 5 specimens) was loaded into the tensile testing fixture of a dynamic mechanical analyzer (DMA, TA Instruments Inc., DE) and subjected to a load of 18N in the ramp force mode [30]. A ramp force of 0.1 N/mm was applied. Displacement was measured with an optical encoder. The stress vs. strain curve was generated and the elastic modulus was determined from the initial 10% strain at the linear regime for each specimen. The stress at maximum from the stress vs. strain curve was taken as the tensile strength of the specimen. The data were reported as average value ± standard deviation.

### Isolation and culture of MSCs

Human MSCs were isolated from bone marrow donations, as previously described [31]. Briefly, cells were pelleted by centrifugation, resuspended in Dulbecco's Modified Eagle Medium (DMEM), and then applied to a Histopaque-1077 column (Sigma, St. Louis, MO). A density gradient was generated by centrifugation at 500 *g* for 30 min. Cells from the DMEM/Histopaque interface were extracted with a syringe and seeded onto tissue culture dishes and cultured in DMEM containing 10% fetal bovine serum. For fluorescent live cell imaging studies, lentivirus-transduced human MSCs constitutively-expressing green fluorescent protein (GFP) were provided by the Tulane Center for Gene Therapy (New Orleans, LA). The GFP-MSCs were selected for stable GFP expression by the vendor. The protocols for isolation and propagation of MSCs were approved by the Univ. of Alabama Institutional Review Board, which determined that our studies qualified for the “No Human Subjects” designation and therefore did not require informed consent (approval # N060810001).

### Scanning Electron Microscopy (SEM) analysis of MSC morphology

MSCs grown on scaffolds for 24 h were fixed in 2.5% glutaraldehyde and then dried in a gradient of ethanol in water, followed by a gradient of hexamethyldisilazane (HMDS) in ethanol. SEM imaging was performed on a Philips 515 SEM with an accelerating voltage of 15 kV.

### Live cell imaging of GFP-labeled MSCs

Scaffolds were placed into sterile 24-well CellCrown™ inserts (Scaffdex, Tampere, Finland) to prevent sample floating or deformation. The effective area of the scaffold when placed in a 24-well CellCrown™ is 0.8 cm^2^. Scaffolds were sterilized in 70% EtOH prior to cell seeding. GFP-labeled MSCs were seeded at a density of 9,000 cells/cm^2^ (∼7,500 cells/scaffold) and cultured in growth media (DMEM containing 4.5 g/L glucose, supplemented with 10% fetal bovine serum) at 37°C, exchanging media every 2–3 days. Visualization of the GFP-expressing cells was performed using a Nikon 80i stereomicroscope.

### MSC Proliferation on electrospun scaffolds

Scaffolds were placed into sterile 48-well CellCrown™ inserts as described above. The effective area of the scaffold when placed in a 48-well CellCrown™ is 0.4 cm^2^. MSCs were seeded at a density of 10,000 cells/cm^2^ and cultured in growth media at 37°C, exchanging media every 2–3 days. At time points of 1 and 4 days, a modified MTS assay (Promega, Madison, WI) was performed to calculate relative cellular proliferation rates. After incubation in media containing MTS reagents, scaffolds were removed from the CellCrown™ and washed in PBS. The scaffolds were then boiled in 1% TX-100 lysis buffer to ensure MTS product was removed from the scaffolds and cells. The supernatant was then read for absorbance at 490 nm on a BioTek Synergy 2 microplate reader. Experiments were performed three separate times, with each independent experiment performed in duplicate, and values were normalized to cell proliferation on PCL scaffolds at day one. Error bars indicate Standard Error of the Mean. Statistical analysis between groups was performed using an unpaired student t-test. Differences were considered significant for probability values less than 0.05.

### Protein adsorption on scaffolds

Scaffolds were either coated overnight with FBS, or placed into rat tibial defects for 30 minutes as previously reported [32]. For the tibial implants, male Sprague-Dawley rats were anesthetized with a 4% isoflurane/oxygen mixture. Under a sterile field, a 1 mm round defect was made in the proximal right tibia, and a scaffold was inserted. After 30 minutes, the scaffolds were retrieved and washed extensively to remove loosely bound proteins. Proteins remaining on the FBS-coated, or tibial-implanted, scaffolds were solubilized in boiling sodium dodecyl sulfate (SDS)-buffer (50 mM Tris, 2% SDS, 5% β-mercaptoethanol) for 30 minutes, with constant agitation. The supernatants were collected and stored at −80°C. Desorbed proteins were resolved on a 7% polyacrylamide gel. Proteins were transferred to a polyvinyldifluoride (PVDF) membrane, and exposed to antibodies for fibronectin (Chemicon AB1954, 1∶1000), or vitronectin (Abcam MAB 1945, 1∶2500); followed by an HRP-conjugated secondary antibody (Amersham Life Sciences, NA934V, 1∶5000, and NA931V, 1∶2500). Proteins were detected using chemiluminescence reagents (Amersham Life Sciences or Millipore).

Experiments involving male Sprague-Dawley rats were carried out in strict accordance with the recommendations in the Guide for the Care and Use of Laboratory Animals of the National Institutes of Health. The protocol was approved by the Institutional Animal Care and Use Committee at the University of Alabama at Birmingham (approval #091107667). Surgeries were performed under isoflurane anesthesia, and all efforts were made to minimize suffering. Two independent runs of this experiment were performed with one animal per scaffold for each run. Western blot images are representative of both runs.

### Immunocytochemical staining for phosphorylated Focal Adhesion Kinase

Scaffold solutions were electrospun onto cover slips in order to create a coating of electrospun nanofibers. Coated cover slips were placed into low-adhesion wells of a 24-well plate and sterilized with 70% EtOH. MSCs were seeded onto substrates at a density of 800 cells/cm^2^ and allowed to adhere for 5 hours. After this time point, cells were fixed in 4% formaldehyde and permeabilized in a 0.1% TX-100 solution. Immunostaining was performed using primary rabbit antibodies against phosphorylated Focal Adhesion Kinase (pFAK Y397, Invitrogen 44-624G, 1∶400 dilution); followed by an Alexa Fluor 568 conjugated secondary anti-rabbit antibody (Invitrogen A10042, 1∶400, dilution). Cells were counterstained with DAPI (Invitrogen 1∶20,000) in order to show cell nuclei.

## Results and Discussion

### Tensile properties of electrospun composites

One of the benefits in mixing PCL with collagen I is that it allows tuning of scaffold tensile strength. This is important in light of recent evidence demonstrating that cells exhibit poor adhesion and survival on matrices that lack sufficient stiffness [33], [34]. Accordingly, we tested the mechanical properties of the four electrospun formulations. The tensile properties of the scaffolds under both dry and wet conditions are given in Table 1. Dry properties provide information regarding durability in surgical handling, whereas wet properties represent the physiological condition experienced by cells. As shown in Table 1, the wet modulus of the PCL, PCL/HA and PCL/col/HA substrates ranged from 13.4–8.4 MPa, and the wet tensile strength was 6.5–2.6 MPa. Notably, the hydrated scaffolds composed of 100% collagen I were markedly more fragile; in fact, scaffolds broke apart immediately upon applying force, therefore values could not be recorded. Similar findings have been reported by Shields et al., claiming that electrospinning of collagen disrupts the natural intermolecular cross-linking of collagen which in turn leads to dissolution of the scaffolds when placed in aqueous solutions [35]. In comparison, blending collagen I with PCL (as represented by the PCL/col/HA scaffolds) significantly increased scaffold strength relative to scaffolds composed of collagen I alone, thus providing a useful alternative to chemical cross-linking of collagen fibers.

**Table 1:** Tensile Properties of dry and hydrated scaffolds.

SCAFFOLD	TENSILE STRENGTH (MPa)		TENSILE MODULUS (MPa)		TENSILE STRAIN (%)	
	Dry	Wet	Dry	Wet	Dry	Wet
PCL	6.5±0.74	6.46±0.34	14.63±0.85	13.37±1.40	73.96±3.5	87.35±3.20
PCL/HA	3.99±0.31	3.03±0.98	9.14±1.15	9.23±1.88	93.82±8.3	51.48±4.2
PCL/col/HA	4.67±0.82	2.62±0.92	13.93±4.94	8.38±0.29	70.64±7.98	75.3±15.69
col	1.46±0.35	---	18.26±3.14	---	20.0±5.0	---

Values represent the average ± standard deviation calculated in the linear portion at 10% strain. The hydrated collagen scaffolds have very low mechanical properties and could not be measured by this technique.

### MSCs adhere and spread on scaffolds composed of PCL, PCL/HA and PCL/col/HA, but not col I

**Figure 1:**
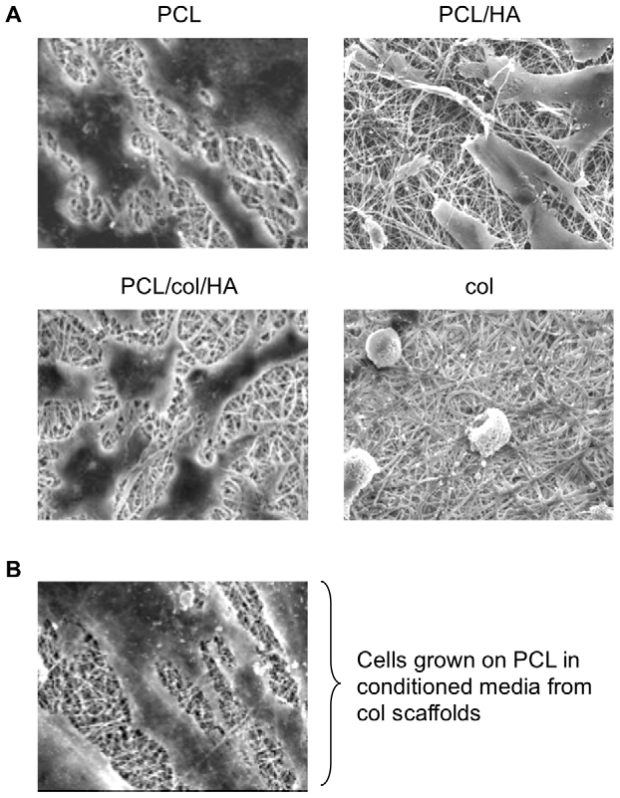
SEM images of MSCs cultured on nanofibrous scaffolds for 24 hours. A) Cell spreading was observed on PCL, PCL/HA, and PCL/col/HA scaffolds, but not on 100% collagen I (col). B) Col scaffolds (without cells) were incubated in culture media for 24 hrs to allow the potential release of soluble factors, and then the solution was collected. MSCs were suspended into this conditioned media, seeded onto PCL scaffolds, and allowed adhere in the media for 24 h. Under these conditions cell spreading was extensive, suggesting that lack of cell spreading on col substrates was not due to any soluble factors released from these scaffolds.

As a first step toward evaluating scaffold cytocompatibility, human MSCs were seeded onto the substrates, and evaluated 24 hrs later for attachment and spreading using scanning electron microscopy (SEM). As shown in Fig. 1A, cells were able to adhere and spread on scaffolds composed of PCL, PCL/HA, and PCL/col/HA, however, MSCs on col scaffolds remained very rounded, suggesting poor cell adhesion. While there are several factors that could account for the lack of cell spreading on col, one possibility was that some inhibitory factor may have been released from the col scaffolds. To test this hypothesis, electrospun col scaffolds (without cells) were incubated in culture media at 37°C to allow for the potential release of soluble factors into the media, and after 24 hours, the media was collected. MSCs were then suspended into this media and seeded onto PCL scaffolds. After 24 hours of adhesion to PCL scaffolds (while in conditioned media from col scaffolds), SEM images were collected (Fig. 1B). These results showed extensive cell spreading, indicating that the poor response of MSCs to the electrospun col scaffolds was not due to any cytotoxic factors released from the substrate.

### Growth of MSCs on scaffolds

**Figure 2:**
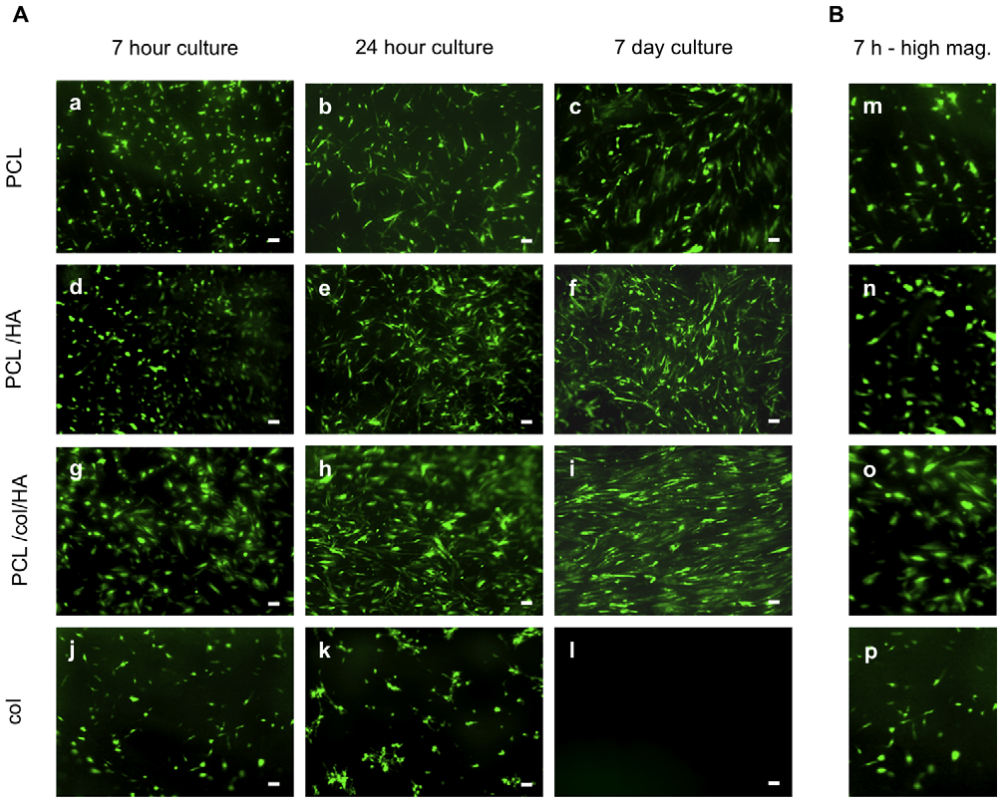
Live cell imaging of GFP-expressing MSCs seeded onto electrospun scaffolds. A) Cells were seeded onto scaffolds and imaged over varying time points. Panels a–c: PCL scaffolds; panels d–f: PCL/HA scaffolds; panels g–i: PCL/col/HA scaffolds and panels j–l: col scaffolds. Scale bar = 100 µm. B) Higher magnification images of GFP-expressing MSCs at seven hours on electrospun scaffolds (panels m–p).

In order to assess cell responses to the scaffolds over more extended culture periods, GFP-expressing MSCs were seeded onto the scaffolds and subjected to live cell imaging at varying time points. The value of this approach is that real-time changes in cell morphology and survival can be monitored on the same samples over the course of their culture, and in addition, using a low magnification allows simultaneous visualization of nearly the entire scaffold surface. Thus, live cell imaging experiments reduce the chance of bias associated with fixing cells at designated time points and then selecting individual representative fields for study. At 7 h following the seeding of GFP-expressing MSCs, adherent cells were apparent on all four of the scaffold formulations (Fig. 2A). The cells adopted a slightly spread morphology on the PCL, PCL/HA and col scaffolds, however spreading was noticeably more extensive on the PCL/col/HA scaffolds at 7 h (see Fig. 2B), suggesting that the tri-component scaffolds provided cells with unique cues that influenced cytoskeletal reorganization. At 24 h, the cells were spread on PCL, PCL/HA and PCL/col/HA scaffolds, and more cells were apparent on the PCL/HA and PCL/col/HA scaffolds as compared with PCL alone. In contrast, only a few very rounded cells, and some cell aggregates, were observed on the col scaffolds at this time point, consistent with the SEM images shown in Fig. 1. At one week following seeding, cells had survived on PCL, PCL/HA and PCL/col/HA scaffolds, although again there appeared to be greater numbers of cells on PCL/HA and PCL/col/HA substrates as compared with PCL. In fact cells were confluent on the PCL/col/HA scaffolds, suggesting that an increased level of proliferation occurred on these substrates. In marked contrast, no cells were apparent on the col scaffolds at one week, reflecting poor survival.

The reason for the lack of cell attachment and survival on col scaffolds is not currently understood. We speculate that this response may be due to the low substrate tensile properties when hydrated. Others have also reported the very low mechanical properties of non-cross-linked electrospun collagen when placed in an aqueous solution, such as cell culture media [18], [35], [36]. A burgeoning literature is revealing that substrate stiffness has a dramatic effect on cell survival and differentiation status [37]. For example, it has been reported that tactile sensing of substrate stiffness by cells feeds back on cell adhesion and cytoskeletal organization [33], [38], and in addition, substrates that are too elastic can cause cell apoptosis [38]. The most common method of increasing the mechanical properties of collagen biomaterials is to use chemical cross-linking agents [16], [17], [35], [36], [39], [40], [41]. However, residual cross-linking agent in the biomaterial has been shown to be cytotoxic [16] and it has been reported that chemical cross-links created by glutaraldehyde, the most common cross-linking agent, can degrade and release cytotoxic aldehydes into the environment [42], [43]. Additionally, Haydarkhan-Hagvall et al. reported that cross-linking of electrospun scaffolds drastically reduces the porosity of the scaffolds, which negatively impacted cell seeding [18]. As an alternative approach to cross-linking, the incorporation of a synthetic polymer to electrospun collagen scaffolds can be used to increase the mechanical properties [18]. Our results clearly show that scaffolds incorporating both PCL and col stimulate greater cell spreading and survival as compared with either PCL or col alone. Elucidating the exact mechanism underlying this result will require future studies, however col substrates were not studied further in the current investigation due to the poor mechanical properties and unfavorable cell responses.

### Cells exhibit greater proliferation on tri-component scaffolds

**Figure 3:**
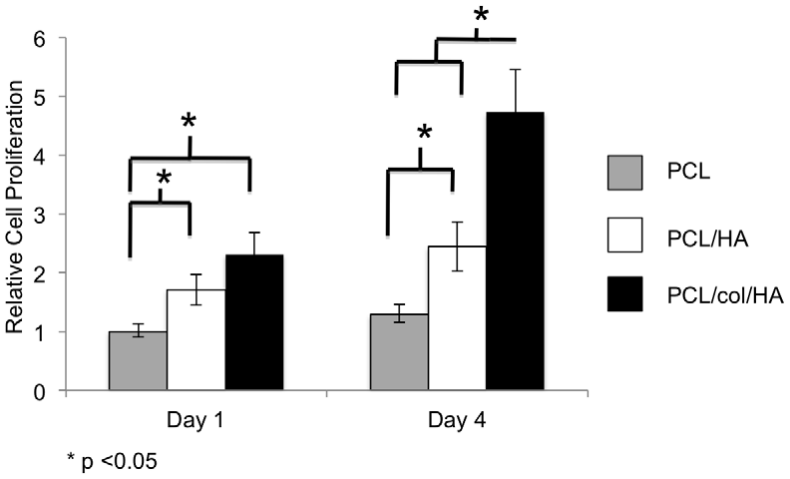
MTS assay quantifying cell proliferation on electrospun scaffolds of PCL, PCL/HA or PCL/col/HA. At day one, cell number was significantly higher on PCL/HA and PCL/col/HA scaffolds in comparison to PCL. By day four, PCL/HA was still significantly higher than PCL, and PCL/col/HA was significantly higher than PCL/HA and PCL. In addition, cell number on PCL/col/HA was significantly higher on day four than day one. An * denotes p<0.05

To quantify the proliferation of MSCs adherent to the scaffolds, an MTS assay was performed (Fig. 3). At 1 day following cell seeding, greater numbers of cells were observed on PCL/col/HA and PCL/HA scaffolds as compared to PCL alone (p<.05), consistent with better cell adhesion to these substrates. At day four, the cell number on PCL/col/HA scaffolds was significantly higher than either PCL or PCL/HA, suggesting that the PCL/col/HA surfaces supported the highest rate of proliferation. MTS is a very common method for monitoring cell proliferation, and is useful because it specifically detects viable cells, in contrast to many other labeling protocols that do not discriminate between live and dead cells. Although it is possible for MTS readings to be influenced by changes in cellular metabolic activity, the MTS results shown in Fig. 3 are in excellent agreement with the GFP-labeled cell imaging studies, which are not influenced by metabolic activity and show that cells are confluent on PCL/col/HA, but not PCL or PCL/HA, scaffolds at 7 days following seeding (Fig. 2).

The quantitative MTS assays lend support for the hypothesis that the addition of col and HA in electrospun scaffolds provides a favorable matrix for MSC attachment and growth. Other groups have seen similar benefits when including collagen or HA in nanofibrous biomaterials. For example, Lee et al. reported significant increases in cellular proliferation of osteoblasts grown on PCL/collagen I electrospun scaffolds compared to PCL alone [44]. Likewise, the addition of HA in PCL electrospun scaffolds by Chuenjitkuntaworn et al. leads to significantly higher levels of primary bone cell growth compared to scaffolds of PCL alone [45]. One of the advances provided by the current study is that both col and HA were incorporated into polymeric electrospun scaffolds, and as previously reported, we were able to minimize agglomeration of the HA particles, thus achieving excellent dispersion of nanoscale HA crystals that approximate the size of native bone HA crystals [28].

### Tri-component scaffolds adsorb greater amounts of adhesion molecules

**Figure 4:**
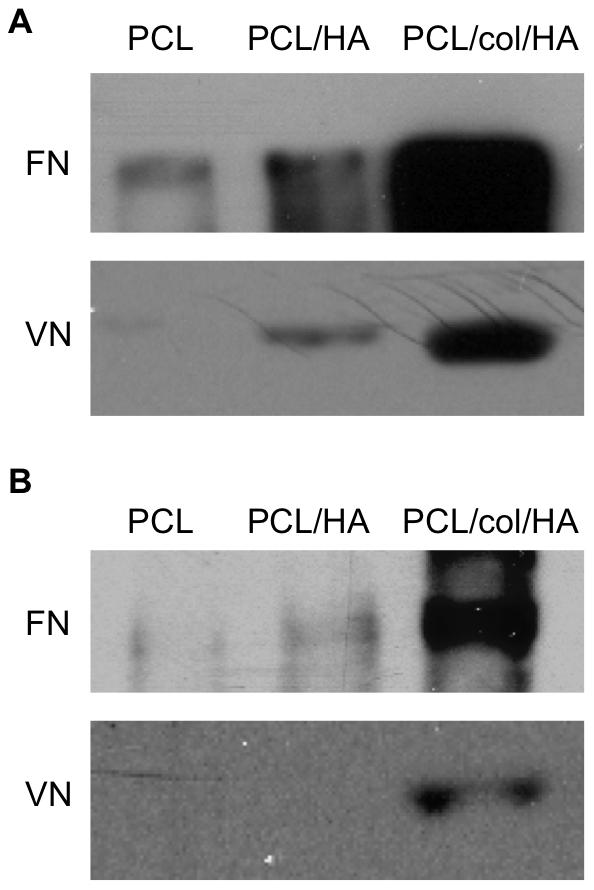
Adsorption of FN and VN by electrospun scaffolds. Scaffolds were coated with fetal bovine serum (A), or implanted into rat tibial osteotomies for 30 min (B). Scaffolds were then washed to remove loosely bound proteins, and proteins were subsequently desorbed by incubation in boiling SDS-containing solution. The amounts of FN and VN were evaluated by Western blot.

The adsorption of bioactive proteins within the tissue microenvironment to the biomaterial surface is known to influence cell/material interactions. This is especially important upon implantation of a biomaterial in a patient, where it is immediately coated with blood and other bodily fluids that contain large amounts of pro-adhesive proteins. Given that HA is known to have a high capacity for protein adsorption, we hypothesized that the incorporation of HA into the scaffolds would increase the amounts of fibronectin (FN) and vitronectin (VN) adsorbed from serum in the media, which in turn would be expected to stimulate integrin-dependent behaviors such as cell adhesion and survival. To test this hypothesis, we monitored the amount of FN and VN bound to the scaffolds following incubation in fetal bovine serum (FBS). Protein adsorption was assessed by Western blot analysis of proteins that were desorbed by incubation in boiling SDS buffer. As shown in Fig. 4A, the PCL/HA scaffolds adsorbed greater amounts of FN and VN from FBS than PCL alone, as expected. However, markedly greater protein adsorption was apparent on PCL/col/HA scaffolds when compared with either of the other two formulations, indicating that the inclusion of collagen I into the scaffolds increased protein adsorption beyond that observed with HA. This is likely due to the fact that col is known to have specific binding interactions with both FN and VN [46], [47]. The enhanced adsorption of FN and VN from serum may have contributed to the increased cell adhesion and proliferation observed on tri-component scaffolds (Figs. 2 and 3).

The adsorption of FN and VN has clinical relevance since implanted biomaterials are immediately exposed to the patient's bodily fluids. Once FN and VN are adsorbed onto a biomaterial, they provide adhesive ligands for MSCs that infiltrate into the wound site. It is well established that FN and VN promote integrin-dependent cell adhesion, survival and proliferation [48] and these molecules have also been implicated in osteoblastic differentiation [14], [15], [49]. To evaluate protein adsorption in a *bona fide* implant site, scaffolds were implanted into rat tibiae for 30 minutes to allow endogenous protein adsorption from the bone microenvironment. As shown in Fig. 4B, substantially greater amounts of FN and VN were bound to the retrieved tri-component scaffolds. Collectively these results suggest that in vivo, tri-component scaffolds will provide a surface rich in integrin-binding proteins, such as col, FN, and VN, that in turn can direct binding of osteogenic cells to the material surface.

### Tri-component scaffolds promote the phosphorylation and activation of Focal Adhesion Kinase

**Figure 5:**
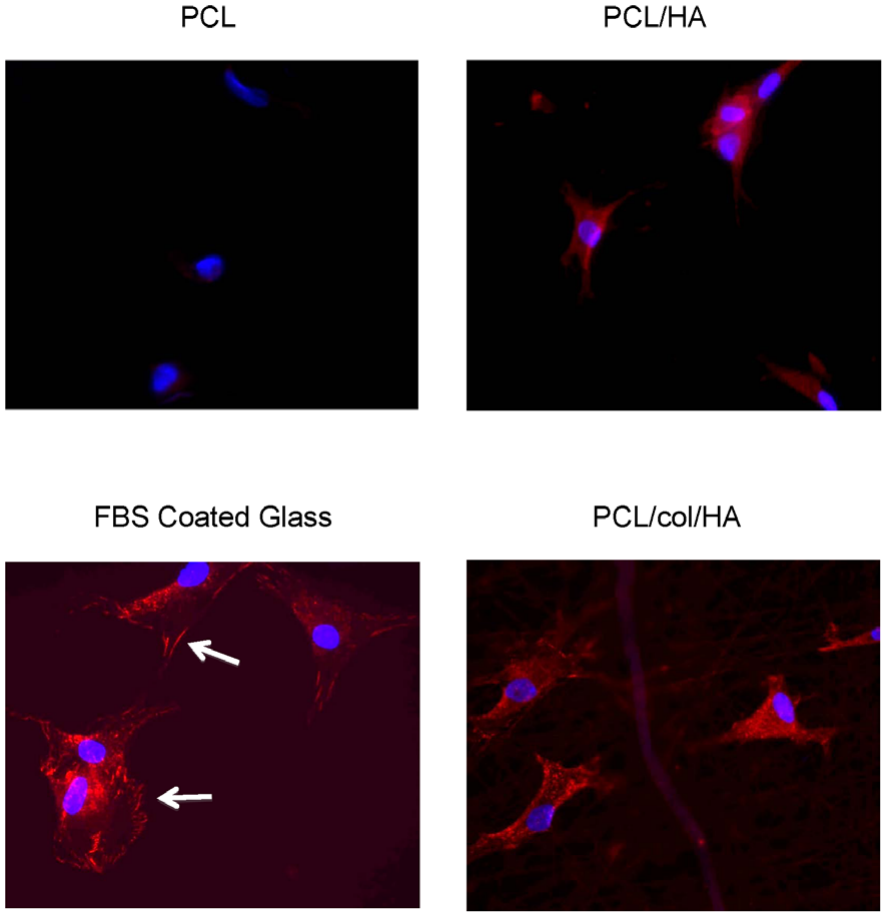
Immunostaining for phosphorylated Focal Adhesion Kinase. MSCs were seeded onto glass coverslips coated with electrospun nanofibers, or with FBS as a control. After 5 hours, cells were fixed and stained for phosphorylated Focal Adhesion Kinase (red). Cells were counterstained with DAPI to show cell nuclei (blue). Cells seeded onto PCL/col/HA scaffolds were better spread, and exhibited greater amounts of punctuate pFAK staining (site pY397) as compared with cells on PCL or PCL/HA. Cells seeded onto FBS-coated glass coverslips displayed pFAK staining in focal adhesion-type structures (white arrows), as expected for cells grown on 2D surfaces.

Anchorage-dependent cells, such as MSCs, rely on the binding of integrins to ligands in order to promote cell survival through downstream signaling cascades. Upon integrin attachment to proteins within the extracellular matrix, one of the early intracellular events to occur is the autophosphorylation of FAK [50]. Activation of FAK, a protein tyrosine kinase, initiates numerous signal transduction pathways that ultimately lead to increased MSC survival and proliferation [15], [51]. To evaluate the capacity of the matrices to induce integrin-associated signaling, MSCs were seeded onto PCL, PCL/HA, or PCL/col/HA scaffolds, and then immunostained for phosphorylated FAK. Cells were also counterstained with DAPI to show cell nuclei. It was apparent that cells seeded on tri-component scaffolds showed markedly increased levels of pFAK, as well as greater cell spreading, as compared with PCL or PCL/HA (Figure 5). Some weak and diffuse cytosolic pFAK staining was evident for cells on PCL/HA scaffolds, but not on PCL. The limited activation of pFAK observed on cells attached to PCL/HA scaffolds may be due to the HA in the scaffolds adsorbing pro-adhesive proteins such as FN and VN from the FBS in the media, as shown previously (Figure 4). As with collagen I, integrin binding to FN and VN induces FAK phosphorylation [52]. Of note, it was observed that the pFAK staining pattern for cells adherent to PCL/col/HA was more punctate than the classic focal adhesion-type staining observed with cells adherent to FBS-coated glass cover slips (Figure 5). These results are consistent with other studies reporting punctate pFAK staining for cells grown in 3-dimensional matrices such as collagen gels [53], rather than 2D tissue culture substrates. The higher levels of FAK phosphorylation observed in cells adherent to PCL/col/HA suggest stronger activation of integrin-dependent signaling cascades, which in turn are important for cell survival and osteoblastic differentiation of MSCs. For example, multiple investigators have shown that the phosphorylation of FAK upon integrin binding leads to activation of the osteogenic transcription factor, Runx2/Cbfa-1, as well as enhanced expression of other osteoblastic markers [52], [54]. Future studies will be focused on examining the capacity of tri-component matrices to induce osteoblastic differentiation of MSCs and in vivo bone regeneration.

### Conclusion

The results presented in this study suggest that tri-component, bone-mimetic, PCL/col/HA scaffolds blend the advantageous mechanical properties of PCL with the favorable biochemical cues provided by the native bone molecules, collagen I and HA. As compared with scaffolds composed of col I, PCL or PCL/HA, tri-component scaffolds supported better cell adhesion, spreading, proliferation and FAK activation. Tri-component scaffolds also adsorbed greater amounts of fibronectin and vitronectin from both serum and the bone microenvironment, thus providing additional ligands for cell surface integrins. Taken together, results from the current study suggest that tri-component PCL/col/HA matrices have high potential to serve as excellent supports for endogenous reparative cells that infiltrate into the implant site, as well as promising substrates for the delivery of exogenously-expanded stem cells.
